# Novel risk genes identified in a genome-wide association study for coronary artery disease in patients with type 1 diabetes

**DOI:** 10.1186/s12933-018-0705-0

**Published:** 2018-04-25

**Authors:** Romain Charmet, Seamus Duffy, Sareh Keshavarzi, Beata Gyorgy, Michel Marre, Peter Rossing, Amy Jayne McKnight, Alexander P. Maxwell, Tarun veer Singh Ahluwalia, Andrew D. Paterson, David-Alexandre Trégouët, Samy Hadjadj

**Affiliations:** 1 Institut National pour la Santé et la Recherche Médicale (INSERM), Unité Mixte de Recherche en Santé (UMR_S) 1166, Team Genomics & Pathophysiology of Cardiovascular Diseases, Sorbonne Universités, UPMC Univ. Paris 06, Paris, France; 2 ICAN Institute for Cardiometabolism and Nutrition, Paris, France; 3 Centre for Public Health, Queen’s University of Belfast, Belfast, Northern Ireland UK; 4 Genetics & Genome Biology Program, Hospital for Sick Children, Toronto, Canada; 5 Départment de Diabétologie, Endocrinologie et Nutrition, Assistance Publique Hôpitaux de Paris, Hôpital Bichat, DHU FIRE, Paris, France; 6 UFR de Médecine, Université Paris Diderot, Sorbonne Paris Cité, Paris, France; 7 Steno Diabetes Center Copenhagen, Gentofte, Denmark; 8 University of Copenhagen, Copenhagen, Denmark; 9 Dalla Lana School of Public Health, University of Toronto, Toronto, Canada; 10 UFR de Médecine et Pharmacie, Université de Poitiers, Poitiers, France; 11 INSERM, CIC 1402 & U1082, Poitiers, France; 12 Service d’Endocrinologie-Diabétologie and Centre d’Investigation Clinique, CHU de Poitiers, BP 577, 86021 Poitiers Cedex, France

**Keywords:** Type 1 diabetes, Coronary artery disease, Diabetic nephropathy, Genome-wide association study, Case control study, Epidemiology, Genetic association studies, Meta-analysis

## Abstract

**Background:**

Patients with type 1 diabetes are more at risk of coronary artery disease than the general population. Although evidence points to a genetic risk there have been no study investigating genetic risk factors of coronary artery disease specific to individuals with type 1 diabetes. To identify low frequency and common genetic variations associated with coronary artery disease in populations of individuals with type 1 diabetes.

**Methods:**

A two-stage genome wide association study was conducted. The discovery phase involved the meta-analysis of three genome-wide association cohorts totaling 434 patients with type 1 diabetes and coronary artery disease (cases) and 3123 T1D individuals with no evidence of coronary artery disease (controls). Replication of the top association signals (p < 10^−5^) was performed in five additional independent cohorts totaling 585 cases and 2612 controls.

**Results:**

One locus (rs115829748, located upstream of the MAP1B gene) reached the statistical threshold of 5 × 10^−8^ for genome-wide significance but did not replicate. Nevertheless, three single nucleotide polymorphisms provided suggestive evidence for association with coronary artery disease in the combined studies: *CDK18* rs138760780 (OR = 2.60 95% confidence interval [1.75–3.85], p = 2.02 × 10^−6^), *FAM189A2* rs12344245 (OR = 1.85 [1.41–2.43], p = 8.52 × 10^−6^) and *PKD1* rs116092985 (OR = 1.53 [1.27–1.85], p = 1.01 × 10^−5^). In addition, our analyses suggested that genetic variations at the *ANKS1A*, *COL4A2* and *APOE* loci previously found associated with coronary artery disease in the general population could have stronger effects in patients with type 1 diabetes.

**Conclusions:**

This study suggests three novel candidate genes for coronary artery disease in the subgroup of patients affected with type 1 diabetes. The detected associations deserve to be definitively validated in additional epidemiological studies.

**Electronic supplementary material:**

The online version of this article (10.1186/s12933-018-0705-0) contains supplementary material, which is available to authorized users.

## Background

Type 1 diabetes (T1D) is a chronic disease characterized by an increase in blood glucose due to a lack of insulin production. Diabetes is a major health concern globally with a prevalence ranging between 4 and 7.8% in industrialized countries [1, 2]; among persons with diabetes, it is estimated that 5–10% are affected with T1D [3]. Recent large-scale epidemiological studies suggest that T1D is associated with a reduced lifespan of approximately 12 years [4] due to at least two-fold increased risk for death due to cardiovascular (CV) events.

Of note, in a nation-based registry of type 1 diabetes, coronary heart disease was 4 times more prevalent in patients with type 1 diabetes compared to sex- and age-matched controls [5].

However, studies exploring cardiovascular disease (CVD) risk variants in T1D patients are lacking. Traditional risk factors for CVD including age, circulating lipid levels, and smoking are well established in the general population [6] and in patients with type 1 diabetes [7]. Coronary artery disease (CAD) could be strongly influenced by genetic factors [8]. However, to the best of our knowledge, the relationship between genetic factors and CAD in type 1 diabetes was largely examined with a candidate gene approach [9–12]. Another important question is whether genetic markers of CAD established in the general population, known to roughly explain 10% of the heritability [13, 14], also play a role in individuals with T1D.

In an attempt to unravel the genetic determinants of CAD among T1D patients, we examined the association of genome-wide genotype array data with CAD in multiple T1D cohorts of European descent (The British Isles, Denmark and France).

## Materials and methods

### General workflow

The present work reports the results of a two-stage research strategy for common genetic variations associated with CAD risk in T1D patients. The first (discovery) stage was based on the meta-analysis of three GWAS cohorts totaling 434 T1D patients with CAD (cases) and 3123 T1D patients with no evidence of CAD (controls). The second stage consisted of a replication of the top discovery signals with association p < 10^−5^ in five additional T1D studies totaling 585 cases and 2612 controls.

### Participating cohorts for the discovery and replication stages

All participants were patients with T1D diagnosed using ADA criteria [15].

Controls were patients with T1D without history of CAD while cases were patients with T1D and a personal history of myocardial infarction or coronary artery revascularization (coronary artery angioplasty or by-pass grafting).

The discovery phase was composed of European-ancestry adults from (1) France (pooled cohorts of T1D from Corbeil Essonnes, Poitiers, Nantes, Paris, Toulouse [16] and two large scale multicenter cohorts i.e. GENESIS/GENEDIAB [16]), (2) Denmark (After-EU cohort [17]) and (3) British Isles (UK-ROI study [18]). The replication phase included five independent T1D cohorts recruited in North America (Additional file 1: Table S1).

All participating studies were approved by their respective institutional review board/ethics committee and an informed consent was obtained from all participating individuals.

### Genotype determinations and imputation

For each participating study, DNA samples were genotyped with high-density SNP arrays and further imputed for SNPs available in the 1000 Genomes reference dataset. Summary descriptions of genotyping technologies, quality control procedures, and used imputation methods (MACH/Impute2) are shown in Additional file 1: Table S1.

### Discovery phase: meta-analysis of discovery GWAS

Association analyses of imputed SNPs with CAD risk were performed separately in each study. Analyses were performed using either of the MACH [19], Quicktest (http://toby.freeshell.org/software/quicktest.shtml), or Plink [20] analyses tools implementing a logistic regression model where the allele dosage representing the expected number of a given reference allele at the imputed SNP was used as covariate to estimate SNP effect. Analyses were adjusted for sex, age, DN status and potential population sub-structure as defined by SNPs derived principal components.

Only SNPs with acceptable imputation quality (r^2^ > 0.3) in the three discovery cohorts and with estimated minor allele frequency (MAF) ≥ 1% were kept for meta-analysis. This was performed by use of a fixed-effects model based on the inverse-variance weighting method as implemented in the METAL software [21]. The statistical threshold (*p* < 5 × 10^−8^) was used for declaring genome-wide statistical significance while controlling for the number of independent tests across the genome. The Cochran’s Q statistic was used to assess heterogeneity of the SNP associations across studies whose magnitude was expressed by the *I*^*2*^ index [22]. Power calculations were performed using the CaTS power calculator (http://csg.sph.umich.edu/abecasis/cats/) [23].

### Replication phase

Similar logistic regression models as those employed in the discovery were used for assessing the association of tested SNPs with CAD risk (Additional file 1: Table S1). Results obtained in the independent replication cohorts were then meta-analyzed using the same methodology as in the discovery step. The Bonferroni threshold corresponding to 0.05 divided by the number of tested SNPs was used to declare statistical replication. Unilateral hypothesis testing was adopted at the replication stage. For SNPs that replicated, a meta-analysis of the combined discovery and replications cohorts was performed to produce a more robust estimate of the effect size.

## Results

A total of 6,728,637 imputed SNPs were tested for association with CAD in 3557 T1D patients made of 434 with CAD cases and 3123 controls in the discovery dataset. The meta-analysis results of the discovery GWAS have been summarized in the Manhattan and QQ plots shown in Additional file 2: Figure S1, Additional file 3: Figure S2, respectively.

One locus at chromosome 5q13.2 reached genome-wide significance (*p* < 5 × 10^−8^) with the lead SNP, rs115829748, located upstream of the *MAP1B* gene. The T allele of this low frequency SNP (MAF ~ 0.04), was associated with an Odds Ratio (OR) of 3.16 [95% confidence interval (CI) 2.18–4.59] (*p *= 1.36 × 10^−9^). No other SNP demonstrated suggestive association with CAD at this locus (Additional file 4: Figure S3).

At the p < ~ 1.0 × 10^−5^ threshold, 20 additional loci demonstrated evidence for suggestive association with CAD with little heterogeneity across cohorts (Table 1). Imputation metrics of the top SNPs are provided in the Additional file 5: Table S2. Therefore, we sought to replicate the top 21 signals in five independent T1D cohorts totaling 585 CAD cases and 2612 controls. Replication was feasible for 17 SNPs while four SNPs (rs34319244, rs373009901, rs143723948, rs571622299) were not properly imputed in the replication stage (Table 1). While none of the 17 SNPs reached the pre-specified Bonferroni threshold of 3.0 × 10^−3^ for positive statistical replication, three were however nominally (p < 0.05) associated with CAD in the replication stage, with genetic effects consistent between the discovery and replication studies (Table 1). Of note, no trend for association was observed (p = 0.279) with the *MAP1B* rs115829748 that came out first in the discovery GWAS and that showed similar allele frequencies in the discovery and replication studies.

**Table 1:** Lead SNPs in discovery and replication

						Discovery						Replication					
CHR	BP	Locus	SNP	Type of variation	EA/NEA^a^	EAF^b^	OR^c^	P^d^	Direction^e^	I^2 f^	P_het_^g^	EAF	OR	P^h^	Direction	I^2^	P_het_
1	205484373	CDK18	rs138760780	Intronic	T/C	0.018	3.48	9.20 10^−6^	+++	0	0.943	0.020	1.88	0.014	++++−	0	0.509
2	97455276	CNNM4	rs116656846	Intronic	A/G	0.023	2.57	9.88 10^−6^	+++	0	0.661	0.028	0.89	0.696	−+−	0	0.876
2	155225182	GALNT13	rs17206992	Intronic	G/A	0.057	2.32	6.33 10^−6^	+++	0	0.817	0.052	0.97	0.580	++−+−	0	0.934
2	177645590	AC092162.1	rs113517532	Intergenic	AGAT/A	0.106	1.75	1.90 10^−6^	+++	0	0.912	0.105	0.86	0.887	+−+−	0	0.658
3	13370674	NUP210	rs73018809	Intronic	T/A	0.024	3.50	1.89 10^−7^	+++	59.1%	0.087	0.022	0.60	0.955	−+	0	0.722
3	103975418	MIR548A3	rs28641753	Intergenic	T/C	0.071	2.10	1.59 10^−7^	+++	0	0.863	0.067	1.02	0.440	−++−	62.70%	0.030
4	6171230	JAKMIP1	rs78031527	Intronic	T/C	0.201	1.70	5.41 10^−6^	+++	0	0.493	0.209	1.13	0.114	++−+−	0	0.622
5	10500646	ROPN1L	rs143537377	Intronic	C/A	0.096	1.88	7.89 10^−6^	+++	1.1%	0.577	0.100	0.85	0.885	−+−+	19.20%	0.292
5	71394387	MAP1B	rs115829748	Intergenic	T/C	0.040	3.16	1.36 10^−9^	+++	71.9%	0.028	0.036	1.13	0.283	−+++−	0	0.655
6	95557471	MANEA-AS1	rs9354144	Intergenic	A/T	0.105	1.71	5.78 10^−6^	+++	0	0.785	0.097	1.05	0.330	−++−	23.90%	0.262
8	73842523	KCNB2	rs571622299	Intronic	A/G	0.015	4.12	6.47 10^−6^	+++	47.2%	0.150	NA	NA	NA	NA	NA	NA
9	37034095	PAX5	rs143723948	UTR5	T/C	0.499	1.65	6.01 10^−7^	+++	0	0.985	NA	NA	NA	NA	NA	NA
9	71955717	FAM189A2	rs12344245	Intronic	G/A	0.035	2.52	9.23 10^−6^	+++	0	0.950	0.038	1.45	0.022	+−+++	0	0.560
10	19457387	ARL5B	rs117826205	Intronic	C/T	0.026	2.72	6.30 10^−6^	+++	0	0.482	0.029	1.11	0.315	−++	5.70%	0.374
11	8080425	TUB	rs61879614	Intronic	C/T	0.048	2.94	2.60 10^−6^	+++	9.4%	0.332	0.046	1.11	0.300	+−	0	0.775
16	2160973	PKD1	rs116092985	Missense (W1399R)	G/A	0.097	1.85	1.72 10^−5^	+++	29.5%	0.242	0.096	1.29	0.026	++++−	0	0.488
17	4328164	SPNS3	rs34319244	Intergenic	C/CT	0.440	1.51	2.59 10^−6^	+++	48.8%	0.142	NA	NA	NA	NA	NA	NA
18	45399356	SMAD2	rs113114656	Intronic	T/C	0.040	2.67	2.30 10^−6^	+++	2.4%	0.359	0.038	1.04	0.437	−++−+	43.40%	0.132
21	21347156	NCRNA00320	rs67213764	Intergenic	G/A	0.261	1.48	9.28 10^−6^	+++	0	0.536	0.260	1.03	0.363	++−	32.20%	0.207
21	24929109	AP000459.7	rs12482425	Intergenic	A/G	0.314	0.66	9.33 10^−6^	−	0	0.509	0.308	1.09	0.871	+++++	0	0.931
22	25988780	ADRBK2	rs373009901	Intronic	C/G	0.019	3.94	9.42 10^−6^	+++	0	0.599	NA	NA	NA	NA	NA	NA

^a^Estimated allele/non estimated allele

^b^Allele frequency of the estimate allele

^c^Odds ratio for disease

^d^Association p value derived from the meta-analysis of the three discovery cohorts

^e^Directionality of the effects across the contributing cohorts

^f^I^2^ statistics for heterogeneity across the contributing cohorts

^g^p value for homogeneity across the contributing cohorts

^h^One sided test p value of association

The strongest association was observed at the *CDK18* locus where, in the replication stage, the rs138760780-T allele, with frequency 0.02, was associated with an increased odds ratio (OR) for disease of 1.88 [1.07–3.31] (p = 0.014). This value has to compare with 3.48 [2.00–6.04] observed in the discovery cohorts. In the combined discovery and replication cohorts, the meta-analyzed OR associated with the rs138760780 T allele was 2.60 [1.75–3.85] (p = 2.02 × 10^−6^) with no heterogeneity across the discovery and replication stage (p = 0.545).

The second suggestive association holds at the *FAM189A2* locus. The rs12344245-G allele found associated with an increased OR of 2.52 [1.68–3.81] in the discovery cohorts also demonstrated a trend for association with the disease in the replication stage, OR = 1.45 [1.01–2.08] (p = 0.022). Combining the discovery and replication study led to a meta-analyzed OR for disease of 1.85 [1.41–2.43] (p = 8.52 × 10^−6^) with no significant evidence for heterogeneity across stage (p = 0.426).

The third suggestive association was observed for the *PKD1* locus. The rs116092985 minor G associated with a 1.85 [1.408–2.44] and a 1.29 [1.00–1.67] increased risk of CAD in the discovery and replication cohorts, respectively. Altogether, the combined statistical evidence for association of the rs116092985-G allele reached p = 1.01 × 10^−5^ (OR = 1.53 [1.27–1.85]) (p = 0.220 for heterogeneity across studies).

### Candidate CAD SNPs

About 90 loci have been found, through GWAS studies, to harbor common susceptibility alleles associated with CAD in the general population. We sought to investigate how these loci associate with CAD in T1D patients. Results of this investigation are summarized in Table 2. From CAD SNPs identified in previous GWAS [13, 14, 24–26], 95 were well imputed in our discovery phase and showed genetic effects with directionality in our T1D populations that were consistent with those previously reported (Table 2). Imputation quality for these 95 SNPs is given in Additional file 6: Table S3. For three SNPs *ANKS1A*_rs17609940, *COL4A2*_rs11838776 and *TOMM40*_rs2075650 (near the *APOE* locus), the statistical evidence for association with the disease was rather strong with p < 5 × 10^−3^ even though it did not achieve multiple testing correction for the number of tested SNPs (~ 5 × 10^−4^ = 0.05/95). For these three SNPs, the amplitude of the genetic association even tended to be stronger in our T1D patients than that previously reported (Table 2). As an illustration, in our discovery T1D population, the *COL4A1* rs11838776-A allele was associated with an OR of 1.33 [1.11–1.61] while the OR reported in the literature was slightly lower (OR = 1.07). Conversely, the association of the polymorphism at the non-coding ANRIL loci on 9p21, that is known to associate the most with CAD among common polymorphisms, showed a very similar association in our T1D patients (OR = 1.16 [0.993–1.362], p = 0.03) compared to that previously reported (OR ~ 1.21).

**Table 2:** Replication of CAD SNPs previously identified in the general population

SNP	CHR	BP	Locus	Published GWAS results			Discovery GWAS in T1D patients					
				EA^a^	EAF^b^	OR^c^	EAF^d^	OR^e^	[95% CI]	P^f^	Direction^g^	Power^h^
rs11206510	1	55496039	PCSK9	T/C	0.848	1.08	0.815	1.08	[0.883–1.324]	0.223	−++	0.150
rs9970807	1	56965664	PPAP2B	C/T	0.915	1.13	0.906	1.21	[0.904–1.625]	0.098	+++	0.380
rs7528419	1	109817192	SORT1	A/G	0.786	1.12	0.784	1.11	[0.918–1.348]	0.138	+++	0.260
rs11810571	1	151762308	TDRKH	G/C	0.849	1.06	0.854	1.05	[0.836–1.316]	0.339	−+−	0.080
rs1892094	1	169094459	ATP1B1	T/C	0.500	0.96	0.529	1.08	[0.919–1.266]	0.822	−++	0.200
rs6700559	1	200646073	DDX59	T/C	0.470	0.96	0.460	1.15	[0.982–1.338]	0.958	+−+	0.520
rs2820315	1	201872264	LMOD1	T/C	0.300	1.05	0.298	0.97	[0.820–0.156]	0.618	−++	0.060
rs17464857	1	222762709	MIA3	T/G	0.861	1.06	0.842	1.12	[0.901–1.405]	0.148	+++	0.250
rs16986953	2	19942473	AK097927	A/G	0.105	1.09	0.075	1.07	[0.799–1.450]	0.314	+++	0.080
rs7567217	2	21303470	APOB	T/C	0.860	1.07	0.858	1.02	[0.811–1.297]	0.415	−+	0.060
rs7568458	2	85788175	GGCX	A/T	0.449	1.06	0.460	1.06	[0.904–1.247]	0.232	+−+	0.140
rs17678683	2	145286559	ZEB2	G/T	0.088	1.10	0.079	1.23	[0.900–1.692]	0.095	+−	0.390
rs2252641	2	145801461	ZEB2	C/T	0.475	1.03	0.450	1.01	[0.867–1.186]	0.430	++−	0.050
rs1250229	2	216304384	FN1	T/C	0.256	1.07	0.260	0.88	[0.730–1.060]	0.911	−	0.390
rs2571445	2	218683154	TNS1	A/G	0.390	1.04	0.388	1.05	[0.894–1.234]	0.275	+−+	0.110
rs1801251	2	233633460	KCNJ13	A/G	0.350	1.05	0.344	1.15	[0.975–1.352]	0.049	++−	0.490
rs7623687	3	49448566	RHOA	A/C	0.855	1.08	0.864	0.96	[0.756–1.212]	0.626	+++	0.060
rs142695226	3	124475201	ITGB5	G/T	0.138	1.07	0.152	0.95	[0.754–1.195]	0.672	−+−	0.090
rs201477372	3	138099161	MRAS	TTTC/T	0.163	1.08	0.155	1.02	[0.829–1.264]	0.411	−+−	0.060
rs12493885	3	153839866	ARHGEF26	C/G	0.886	1.07	0.866	0.87	[0.691–1.089]	0.890	+−	0.260
rs17087335	4	57838583	NOA1	T/G	0.210	1.06	0.177	0.99	[0.812–1.215]	0.523	−++	0.050
rs10857147	4	81181072	FGF5	T/A	0.275	1.05	0.282	1.02	[0.855–1.215]	0.417	++−	0.060
rs7678555	4	120909501	MAD2L1	C/A	0.301	1.05	0.287	0.92	[0.770–1.100]	0.818	−+	0.200
rs4593108	4	148281001	MIR548G	C/G	0.795	1.07	0.831	0.93	[0.761–1.145]	0.743	+−	0.110
rs1878406	4	148393664	EDNRA	C/T	0.844	0.94	0.894	0.94	[0.740–1.208]	0.328	−+	0.070
rs72689147	4	156639888	GUCY1A3	G/T	0.817	1.07	0.812	1.13	[0.919–1.390]	0.122	+++	0.300
rs273909	5	131667353	SLC22A4	G/A	0.117	1.06	0.104	0.94	[0.726–1.217]	0.680	−	0.090
rs246600	5	142516897	ARHGAP26	T/C	0.480	1.05	0.486	0.98	[0.839–1.155]	0.575	−+−	0.060
rs9349379	6	12903957	PHACTR1	G/A	0.432	1.14	0.392	1.07	[0.919–1.267]	0.174	+++	0.200
rs7454157	6	12909874	PHACTR1	G/A	0.651	1.10	0.622	1.15	[0.978–1.371]	0.044	+++	0.550
rs6909752	6	22612629	HDGFL1	A/G	0.351	1.05	0.374	1.01	[0.851–1.186]	0.476	−+−	0.050
rs3130683	6	31888367	C2	T/C	0.860	1.09	0.960	0.69	[0.455–1.034]	0.964	+−	0.580
rs17609940	6	35034800	ANKS1A	G/C	0.824	1.03	0.796	1.32	[1.077–1.635]	0.004	+++	0.890
rs56336142	6	39134099	KCNK5	T/C	0.807	1.07	0.786	1.04	[0.860–1.265]	0.333	+−	0.080
rs10947789	6	39174922	KCNK5	T/C	0.775	1.05	0.753	1.07	[0.890–1.293]	0.229	+−+	0.150
rs12202017	6	134173151	TCF21	A/G	0.700	1.07	0.713	1.02	[0.856–1.215]	0.411	+−	0.060
rs12190287	6	134214525	TCF21	C/G	0.617	1.06	0.633	1.06	[0.896–1.270]	0.232	+−	0.150
rs2048327	6	160863532	SLC22A3	T/C	0.646	0.94	0.633	1.00	[0.854–1.188]	0.538	−+−	0.050
rs3798220	6	160961137	LPA	T/C	0.975	0.70	0.986	0.74	[0.397–1.393]	0.178	+−	0.140
rs55730499	6	161005610	LPA	T/C	0.056	1.37	0.079	1.22	[0.925–1.609]	0.078	+0+	0.350
rs4252185	6	161123451	PLG	C/T	0.060	1.34	0.087	1.15	[0.862–1.541]	0.168	+++	0.210
rs4252120	6	161143608	PLG	T/C	0.740	1.03	0.709	0.91	[0.766–1.084]	0.852	−+	0.240
rs2023938	7	19036775	HDAC9	T/C	0.897	0.94	0.899	0.87	[0.679–1.135]	0.161	+−	0.200
rs2107595	7	19049388	HDAC9	A/G	0.200	1.08	0.174	1.14	[0.926–1.406]	0.106	+++	0.310
rs12539895	7	107091849	COG5	C/A	0.807	1.04	0.788	1.14	[0.931–1.394]	0.101	+−+	0.350
rs10953541	7	107244545	BCAP29	C/T	0.783	1.05	0.750	1.16	[0.967–1.401]	0.054	++−	0.490
rs11556924	7	129663496	ZC3HC1	C/T	0.687	1.08	0.593	0.97	[0.832–1.147]	0.610	−+	0.060
rs10237377	7	139757136	PARP12	T/G	0.350	0.95	0.362	0.91	[0.766–1.084]	0.148	−+−	0.260
rs3918226	7	150690176	NOS3	T/C	0.060	1.14	0.088	0.90	[0.672–1.212]	0.751	−++	0.140
rs264	8	19813180	LPL	G/A	0.853	1.06	0.846	0.94	[0.756–1.168]	0.711	−+	0.100
rs2954029	8	126490972	TRIB1	A/T	0.551	1.04	0.529	1.05	[0.896–1.232]	0.269	++−	0.110
rs3217992	9	22003223	CDKN2BAS1	C/T	0.607	0.88	0.614	0.92	[0.789–1.084]	0.169	−	0.200
rs2891168	9	22098619	CDKN2BAS1	G/A	0.489	1.21	0.505	1.16	[0.993–1.362]	0.030	+++	0.6
rs2519093	9	136141870	ABO	T/C	0.191	1.08	0.186	0.98	[0.800–1.203]	0.571	−++	0.060
rs2487928	10	30323892	KIAA1462	A/G	0.418	1.06	0.458	1.08	[0.924–1.271]	0.161	−++	0.220
rs2624695	10	44549767	CXCL12	C/T	0.534	0.94	0.503	1.08	[0.926–1.270]	0.843	+++	0.220
rs501120	10	44753867	CXCL12	T/C	0.813	1.08	0.863	1.18	[0.927–1.508]	0.088	+++	0.400
rs11203043	10	90989279	LIPA	G/A	0.576	1.04	0.551	0.89	[0.764–1.052]	0.909	−	0.360
rs1412444	10	91002927	LIPA	T/C	0.369	1.07	0.326	0.94	[0.798–1.116]	0.749	−+−	0.130
rs11191416	10	104604916	CYP17A1	T/G	0.873	1.08	0.915	0.90	[0.686–1.186]	0.768	+−	0.120
rs11042937	11	10745394	MRVI1-CTR9	T/G	0.490	1.04	0.499	1.06	[0.908–1.248]	0.220	++−	0.150
rs3993105	11	13303071	ARNTL	T/C	0.704	1.05	0.693	1.03	[0.866–1.228]	0.364	−+−	0.070
rs12801636	11	65391317	PCNX3	A/G	0.230	0.95	0.225	0.98	[0.810–1.189]	0.425	+−	0.060
rs590121	11	75274150	SERPINH1	T/G	0.300	1.05	0.295	0.80	[0.669–0.961]	0.992	−	0.830
rs9319428	13	28973621	FLT1	A/G	0.314	1.04	0.289	1.03	[0.874–1.227]	0.339	−++	0.070
rs4773144	13	110960712	COL4A2	A/G	0.572	0.95	0.555	0.99	[0.850–1.172]	0.493	−++	0.050
rs11838776	13	111040681	COL4A2	A/G	0.263	1.07	0.284	1.33	[1.113–1.606]	0.001	++−	0.980
rs9515203	13	111049623	COL4A2	T/C	0.761	1.07	0.736	1.20	[0.983–1.468]	0.036	+++	0.650
rs10139550	14	100145710	HHIPL1	G/C	0.423	1.06	0.413	0.96	[0.819–1.145]	0.645	−+	0.070
rs6494488	15	65024204	RBPMS2	G/A	0.180	0.95	0.151	1.01	[0.803–1.280]	0.545	+−	0.050
rs56062135	15	67455630	SMAD3	C/T	0.790	1.07	0.758	1.07	[0.893–1.302]	0.214	++−	0.160
rs7173743	15	79141784	ADAMTS7	T/C	0.564	1.08	0.515	1.00	[0.855–1.171]	0.495	−++	0.050
rs8042271	15	89574218	ABHD2	G/A	0.900	1.10	0.947	0.93	[0.602–1.457]	0.613	−+	0.070
rs17514846	15	91416550	FURIN-FES	A/C	0.440	1.05	0.461	1.08	[0.924–1.267]	0.163	++−	0.210
rs1800775	16	56995236	CETP	C/A	0.510	1.04	0.524	0.99	[0.844–1.153]	0.568	−+−	0.050
rs1050362	16	72130815	DHX38	A/C	0.380	1.04	0.351	1.11	[0.939–1.313]	0.110	+−+	0.310
rs7500448	16	83045790	CDH13	A/G	0.752	1.06	0.755	1.09	[0.894–1.321]	0.202	+−+	0.190
rs216172	17	2126504	SMG6	C/G	0.350	1.05	0.368	1.13	[0.963–1.329]	0.067	+++	0.420
rs12936587	17	17543722	RAI1	G/A	0.611	1.03	0.542	1.00	[0.857–1.174]	0.480	−++	0.050
rs17608766	17	45013271	GOSR2	C/T	0.140	1.07	0.133	0.89	[0.705–1.121]	0.840	−	0.220
rs999474	17	46987665	UBE2Z	G/A	0.600	1.04	0.572	0.92	[0.789–1.084]	0.832	−	0.130
rs7212798	17	59013488	BCAS3	C/T	0.150	1.08	0.155	0.98	[0.786–1.221]	0.570	−++	0.060
rs1867624	17	62387091	PECAM1	C/T	0.390	0.96	0.377	1.06	[0.898–1.241]	0.744	+++	0.130
rs663129	18	57838401	U4/MC4R	A/G	0.260	1.06	0.237	1.01	[0.847–1.227]	0.418	++−	0.060
rs1122608	19	11163601	LDLR	G/T	0.770	1.07	0.756	0.87	[0.731–1.048]	0.926	+−	0.380
rs56289821	19	11188247	LDLR	G/A	0.900	1.14	0.880	1.10	[0.855–1.427]	0.223	+−+	0.170
rs12976411	19	32882020	ZNF507	A/T	0.910	1.61	0.958	0.96	[0.639–1.460]	0.564	−+	0.050
rs8108632	19	41854534	TGFB1	T/A	0.488	1.05	0.445	1.11	[0.938–1.307]	0.113	++−	0.330
rs2075650	19	45395619	TOMM40	A/G	0.865	0.93	0.869	0.74	[0.596–0.919]	0.003	−	0.870
rs445925	19	45415640	APOE/APOC1	G/A	0.902	1.09	0.893	0.87	[0.681–1.129]	0.844	++−	0.210
rs4420638	19	45422946	APOE/APOC1	G/A	0.166	1.10	0.171	1.20	[0.985–1.461]	0.034	+−+	0.530
rs1964272	19	46190268	SNRPD2	G/A	0.510	1.05	0.509	0.95	[0.805–1.113]	0.747	−+	0.110
rs867186	20	33764554	PROCR	G/A	0.110	0.93	0.094	1.26	[0.974–1.623]	0.961	+++	0.490
rs28451064	21	35593827	KCNE2	A/G	0.121	1.14	0.126	1.36	[1.066–1.751]	0.006	+−+	0.890
rs180803	22	24658858	POM121L9P	G/T	0.970	1.20	0.979	1.18	[0.510–2.743]	0.348	−++	0.120

^a^Estimated allele/non estimated allele

^b^Allele frequency of the estimate allele reported in [14, 24–26]

^c^Odds ratio for CAD reported in [14, 24–26]

^d^Allele frequency of the estimated allele in the discovery GWAS of T1D patients

^e^Odds ratio for CAD [95% confidence interval] observed in the discovery GWAS of T1D patients

^f^One sided test p value of association

^g^Directionality of the effects across the contributing cohorts

^h^Power estimates were provided by the CaTS program [23] and correspond to the power of our discovery GWAS to achieve 0.05 statistical significance at the observed associations based on EAF^d^ and OR^e^ under the assumption of a multiplicative model (on log-scale)

## Discussion

The present work was aimed at identifying susceptibility alleles for CAD risk in patient population of T1D using a GWAS approach with a two-step framework (discovery + replication). Albeit we identified one locus (*MAP1B*) reaching genome-wide significance in the discovery stage, it did not replicate with similar effects. Nevertheless, in the combined T1D dataset of 1019 cases and 5735 controls, we observed strong statistical evidence for association with CAD at three biological candidate genes, *CDK18, PKD1* and *FAM189A2*.

We consider our study as very original as it is the first one, to the best of our knowledge, to use a GWAS approach for CAD, in patients with type 1 diabetes. We have thus pooled the largest collection of type 1 diabetes patients with available genetic data.

The low frequency *CDK18* rs138760780-T allele (frequency ~ 0.02) was found associated with ~ 2.5 fold increased risk of CAD. According to public database (e.g. Haploreg [27]), this SNP does not show strong linkage disequilibrium (LD) (pairwise r^2^ > 0.80) with other SNPs at this locus, consistent with the regional association plot that does not suggest any evidence of disease associated SNPs (Additional file 7: Figure S4). Interrogating the functional status of this SNP through HaploReg tool [27] suggested that this SNP may be involved in some epigenetic regulatory mechanisms. *CDK18* encodes for a cyclin-dependent kinase, suggesting a role in cell cycle. This predicted protein is also related to *CDK1*, which is involved in the G2/M transition in eukaryotic cells [28]. Although cell cycle is a very broad pathway, *CDK1* has also been associated with T1D [29], but at this point little is known about a potential involvement of *CDK18* in the pathophysiology of T1D or its complications.

We also observed some evidence that the low frequency *FAM189A2* rs12344245 G allele, (frequency ~ 0.04), associated with a ~ 1.8 fold-increased risk of CAD. We did not find any evidence suggesting that this intronic SNP, or any other SNPs in LD (Additional file 8: Figure S5: regional association plot) with it, could be functional. Nevertheless, even though not much is known about the role of the encoded protein, this locus is a good candidate. Indeed, genetic variations at this locus have been found associated with albumin to creatinine ratio [30]. More interestingly, two *FAM189A2* SNPs (rs10780297 and rs10120442) have been reported to moderately associate (p = 9.3 × 10^−4^) in a large GWAS for CAD in ~ 63,000 non-diabetic populations [14], suggesting that this locus could be a CAD locus in some specific at-risk groups of diabetic patients. The latter two SNPs are in moderate LD (D’ = 1 but r^2^ = 0.05) with our lead rs12344245 SNP, indicating that a fine mapping analysis of this locus would warrant further investigations. Of interest, it was not identified as a common gene in both type 2 diabetes and CAD. It can thus be speculated this gene is an important gene in high-glucose environment rather than a gene leading to high-glucose.

Finally, we observed an association of the non-synonymous *PKD1* rs116092985 (Trp1399Arg) with CAD among T1D patients where the Arg1399 minor allele (frequency ~ 0.10), was associated with an increased CAD risk (OR ~ 1.5). The regional plot (Additional file 9: Figure S6) shows that there are several SNPs in LD with this *PKD1* top SNP that associate with CAD. PKD1 encodes for the Polycystin 1, Transient Receptor Potential Channel Interacting protein, a member of the polycystin protein family. Recent reports have suggested a role of *PKD1* not only in renal tubular function and structure [31] but rare mutations in this gene as the main also cause underlying polycystic kidney disease [32], highlighting its importance in kidney complications. One important question is whether *PKD1* risk allele is involved in a common genetic background linking DN and CAD. This question was not duly analyzed due to power issue. However, no clear association was established with DN in previous GWAS focusing on this question [18].

Our study also enabled us to assess in patients with T1D the impact of common SNPs that have been found associated with CAD in large GWAS performed in unselected individuals. Beyond the observation that most of the previously reported SNPs showed consistent association with CAD in our T1D population, this look-up identified a few CAD loci (*ANKS1A*, *COL4A2*, *TOMM40/APOE*) where the reported CAD associated SNP could have a stronger effect in T1D patients. However, this hypothesis would require further investigation.

Some limitations must be acknowledged. We did not consider differently T1D patients with and without DN, and all of the analyses were not stratified on DN status in order to keep all CAD patients in the analysis. However, this should have limited impact on our main results as none of the SNPs identified here were positive considering previously reported GWAS with regard to DN as primary endpoint [18]. Another limitation pertains to limited power of our sample size required, particular to overcome the harsh genome-wide statistical significance threshold. Indeed, our discovery GWAS was not well powered to identify common SNPs associated with moderate genetic effects as those frequently encountered in a GWAS context. For instance, our discovery study had no power to detect at the genome-wide statistical threshold the genetic effect of a variant with an associated allelic OR less than 1.40. It was only well powered (> 80%) to detect OR greater than 1.6 as soon as the allele frequency of the disease allele is greater than 0.28 and well powered to detect OR greater than ~ 2 for allele frequency greater than 0.05. In particular, we had no power to detect the well-established association of the 9p21 locus at the 5 × 10^−8^ threshold while we had a chance of 60% to detect it would the liberal threshold of 0.05 had been used. Similarly, we acknowledge the low power of our replication studies where none of the tested associations achieved the Bonferroni threshold of 3 × 10^−3^. We only had a power of 53, 38 and 26% to detect at this threshold a significant association at the *CDK18* rs13876070, *PKD1* rs116092985 and the *FAM189A2* loci, respectively.

Despite these limitations, we have assembled the largest cohort available and conducted novel analyses to discover novel candidate loci for CAD in T1D patients that need to be further studied with additional epidemiological data and functional work to confirm our findings. Of interest, our negative study could suggest a role of epigenetics beyond genomics. In this regard, insulin promotes the expression of DNA methyltransferases leading to methylation resulting in atherosclerosis [33], broadening the research field of CAD in type 1 diabetes.

## Conclusions

We identified three new candidate loci for CAD in individuals with T1D, these loci weren’t previously found associated with CAD in the general population. Three other loci previously reported in the general population were found associated with CAD in our setting, namely *ANKS1A*, *COL4A2* and *TOMM40/APOE*. Although this work needs further investigation, studying the function of these loci could lead to a better understanding of the physiological pathways involved in the development of CAD as a complication of T1D.

## Additional files

**Additional file 1: Table S1.** Main design and sample characteristics of the discovery and replication studies.

**Additional file 2: Figure S1.** Quantile-Quantile plot representation of the discovery meta-GWAS results.

**Additional file 3: Figure S2.** Manhattan plot representation of the discovery meta-GWAS results.

**Additional file 4: Figure S3.** Regional association plot at the *MAP1B* locus.

**Additional file 5: Table S2.** Imputation quality of SNPs with association p-values < 1.0 × 10^-5^ in the discovery cohorts.

**Additional file 6: Table S3.** Imputation quality of the established CAD associated SNPs in the discovery cohorts.

**Additional file 7: Figure S4.** Regional association plot at the *CKD18* locus.

**Additional file 8: Figure S5.** Regional association plot at the *FAM189A2* locus.

**Additional file 9: Figure S6.** Regional association plot at the *PKD1* locus.

## Abbreviations

CAD: coronary artery disease
T1D: type 1 diabetes
GWAS: genome wide association study
CVD: cardiovascular disease
DN: diabetic nephropathy
SNP: single nucleotide polymorphism
MAF: minor allele frequency
OR: odds ratio
LD: linkage disequilibrium
CI: confidence interval
