# Supplementary figures and images for: Novel risk genes identified in a genome-wide association study for coronary artery disease in patients with type 1 diabetes
Source: Cardiovasc Diabetol. 2018 Apr 25;17:61. doi: 10.1186/s12933-018-0705-0 (PMC5916834; doi:10.1186/s12933-018-0705-0)

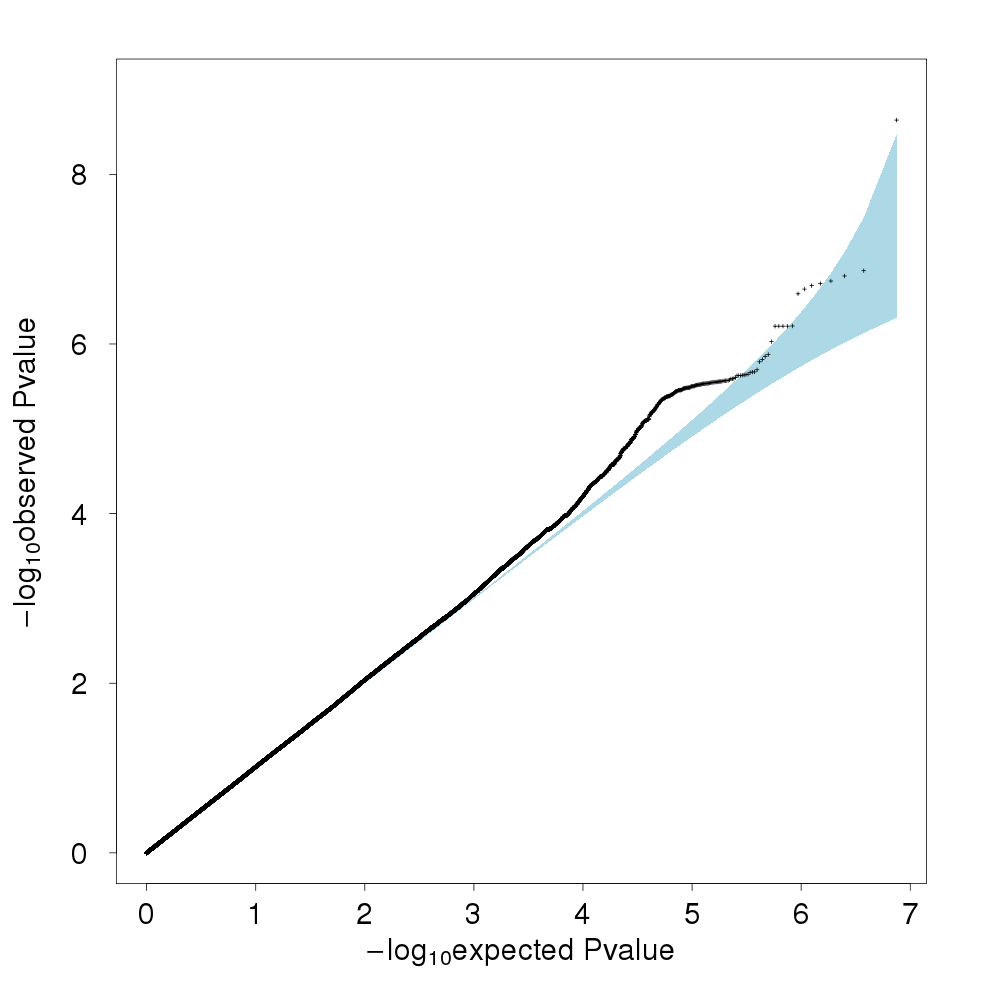

Supplement: Supplementary file 2 — Additional file 2: Figure S1. Quantile-Quantile plot representation of the discovery meta-GWAS results. [file 12933_2018_705_MOESM2_ESM.png]

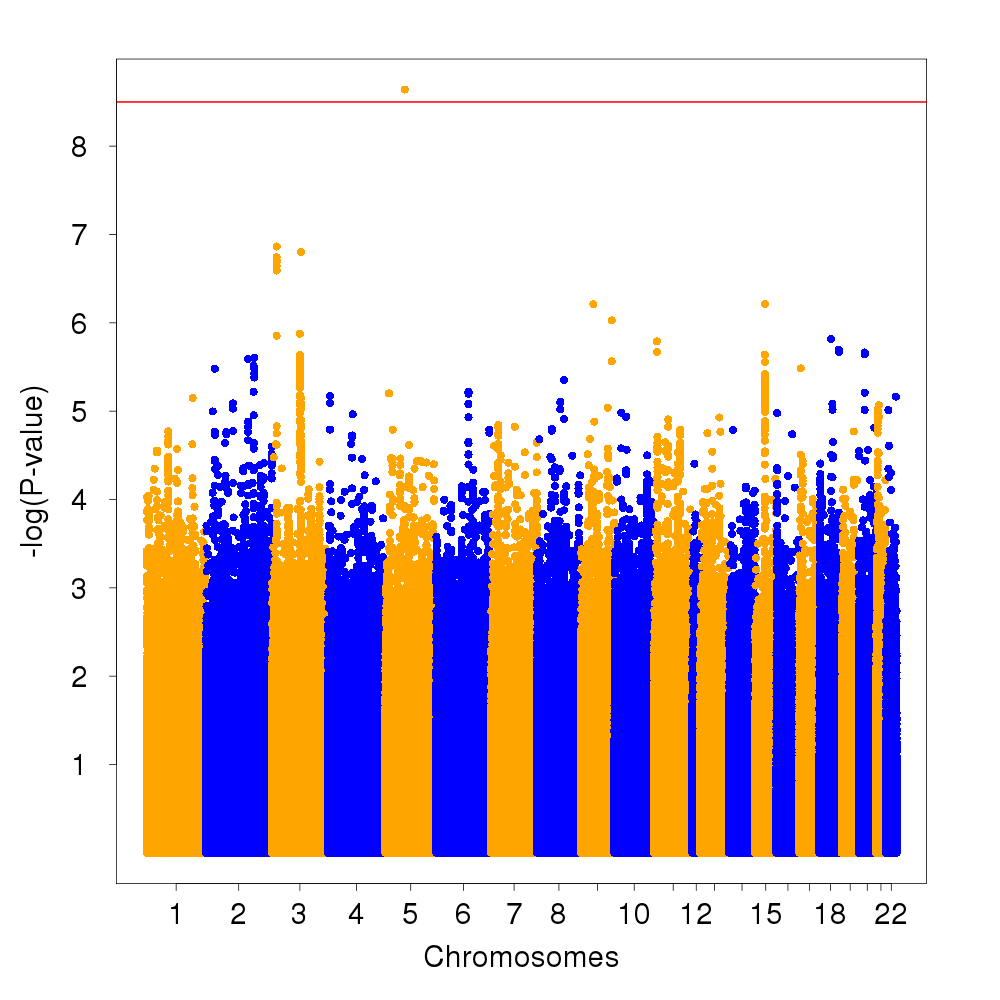

Supplement: Supplementary file 3 — Additional file 3: Figure S2. Manhattan plot representation of the discovery meta-GWAS results. [file 12933_2018_705_MOESM3_ESM.png]
