# Supplementary material for: Novel risk genes identified in a genome-wide association study for coronary artery disease in patients with type 1 diabetes
Source: Cardiovasc Diabetol. 2018 Apr 25;17:61. doi: 10.1186/s12933-018-0705-0 (PMC5916834; doi:10.1186/s12933-018-0705-0)
Supplement: Supplementary file 4 — Additional file 4: Figure S3. Regional association plot at the MAP1B locus. [file 12933_2018_705_MOESM4_ESM.pdf]

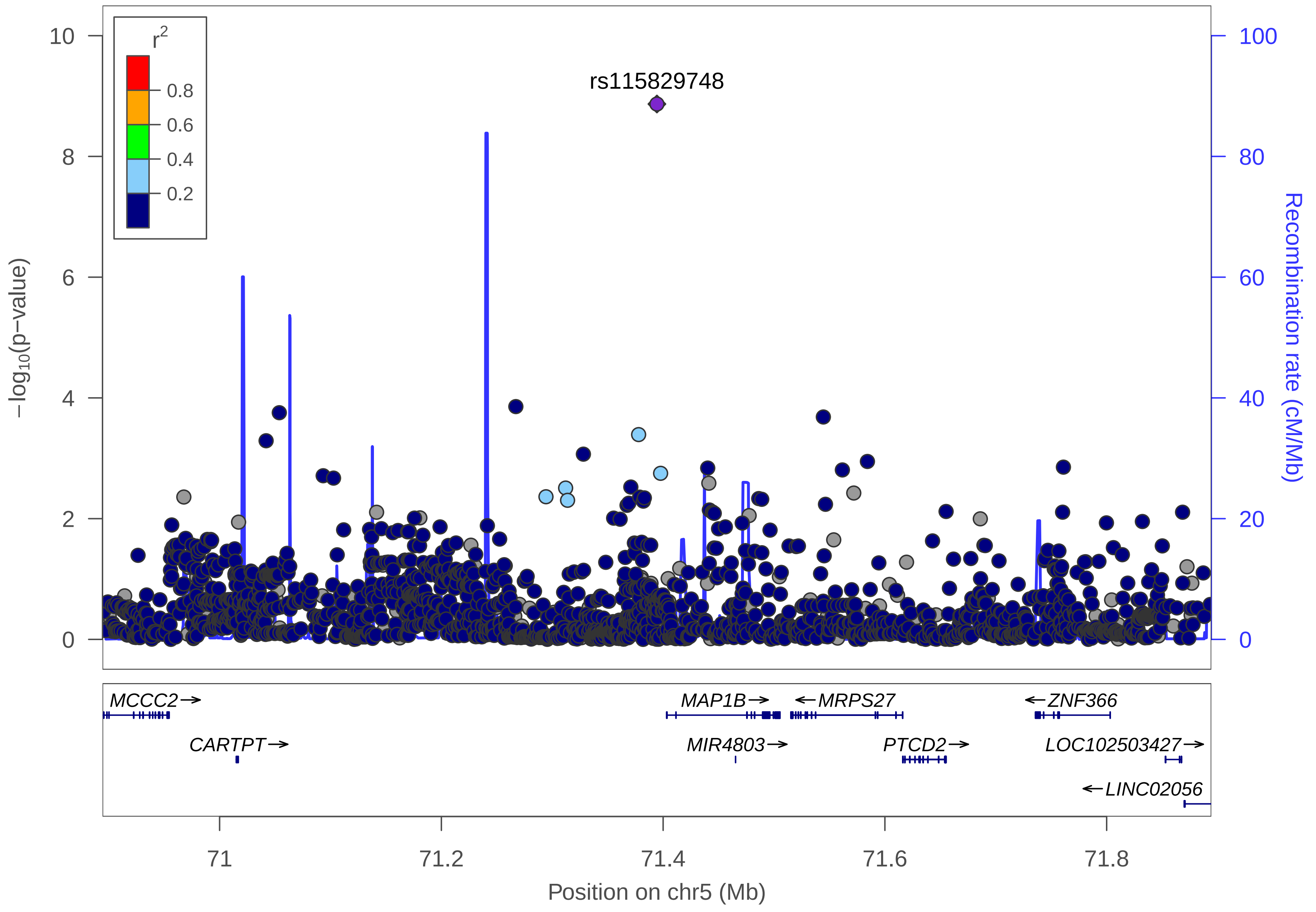

rs115829748
r2
0.8
0.6
0.4
0.2
−log10(p−value)
Recombination rate (cM/Mb)
0
2
4
6
8
10
0
20
40
60
80
100
MCCC2
CARTPT
MAP1B
MIR4803
MRPS27
PTCD2
ZNF366
LOC102503427
LINC02056
71
71.2
71.4
71.6
71.8
Position on chr5 (Mb)

date:	Mon Aug 28 19:52:41 2017
build:	hg19
display range:	chr5:70894387−71894387 [70894387−71894387]
hilite range:	0 − 0 [0 − 0]
reference SNP:	chr5:71394387
number of SNPs plotted:	2315
min P−value:	1.35E−9 [chr5:71394387]
max P−value:	9.99E−1 [chr5:71381885]

Make more plots at http://csg.sph.umich.edu/locuszoom/
