# Supplementary material for: Novel risk genes identified in a genome-wide association study for coronary artery disease in patients with type 1 diabetes
Source: Cardiovasc Diabetol. 2018 Apr 25;17:61. doi: 10.1186/s12933-018-0705-0 (PMC5916834; doi:10.1186/s12933-018-0705-0)
Supplement: Supplementary file 7 — Additional file 7: Figure S4. Regional association plot at the CKD18 locus. [file 12933_2018_705_MOESM7_ESM.pdf]

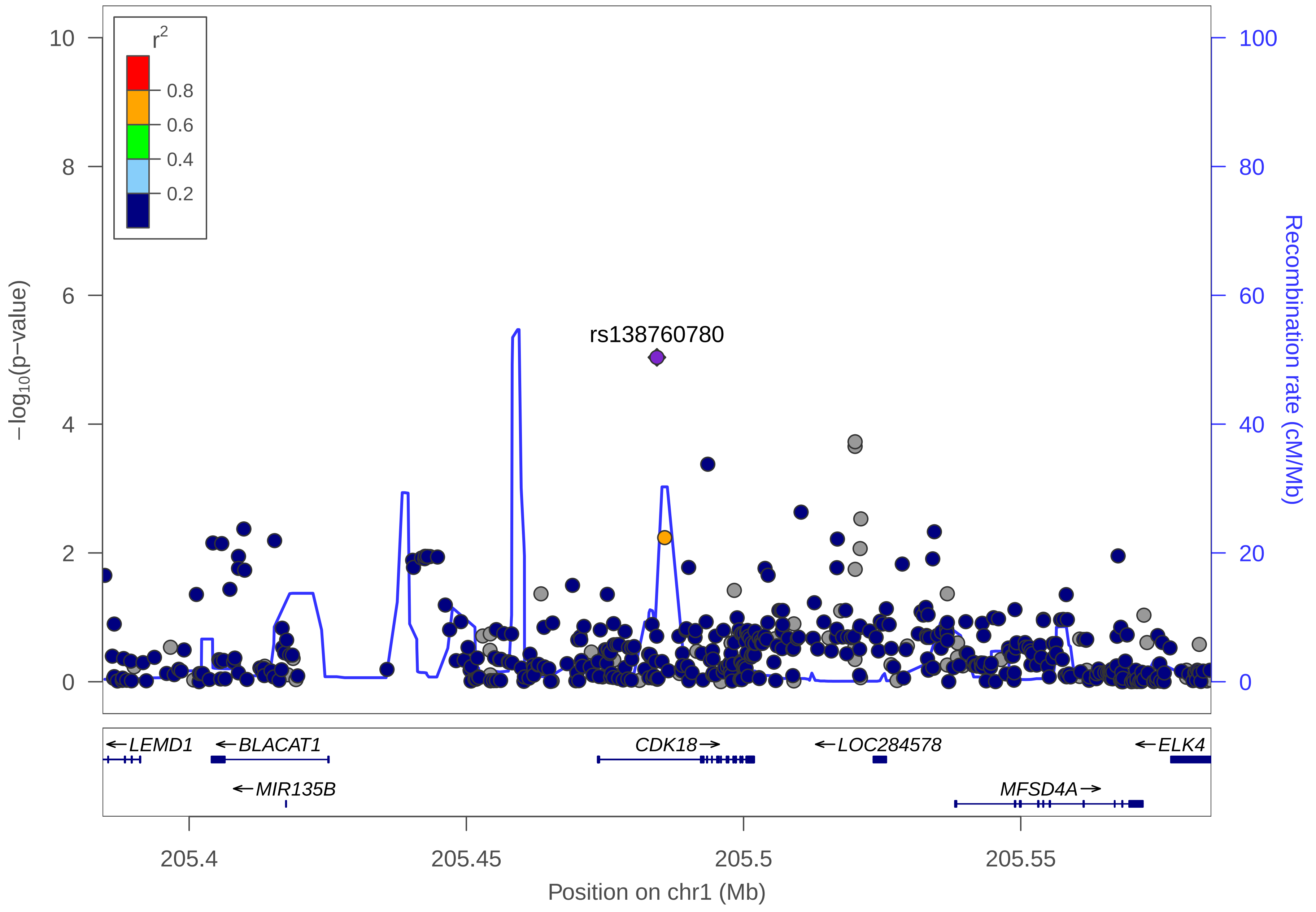

r2
0.8
0.6
0.4
0.2
10
8
6
4
2
0
−log10(p−value)
100
80
60
40
20
0
Recombination rate (cM/Mb)
rs138760780
LEMD1
BLACAT1
CDK18
LOC284578
ELK4
MIR135B
MFSD4A
205.4
205.45
205.5
205.55
Position on chr1 (Mb)

date:	Mon Aug 28 19:27:20 2017
build:	hg19
display range:	chr1:205384373−205584373 [205384373−205584373]
hilite range:	0 − 0 [0 − 0]
reference SNP:	chr1:205484373
number of SNPs plotted:	517
min P−value:	9.2E−6 [chr1:205484373]
max P−value:	10E−1 [chr1:205568438]

Make more plots at http://csg.sph.umich.edu/locuszoom/
