# Supplementary material for: Novel risk genes identified in a genome-wide association study for coronary artery disease in patients with type 1 diabetes
Source: Cardiovasc Diabetol. 2018 Apr 25;17:61. doi: 10.1186/s12933-018-0705-0 (PMC5916834; doi:10.1186/s12933-018-0705-0)
Supplement: Supplementary file 8 — Additional file 8: Figure S5. Regional association plot at the FAM189A2 locus. [file 12933_2018_705_MOESM8_ESM.pdf]

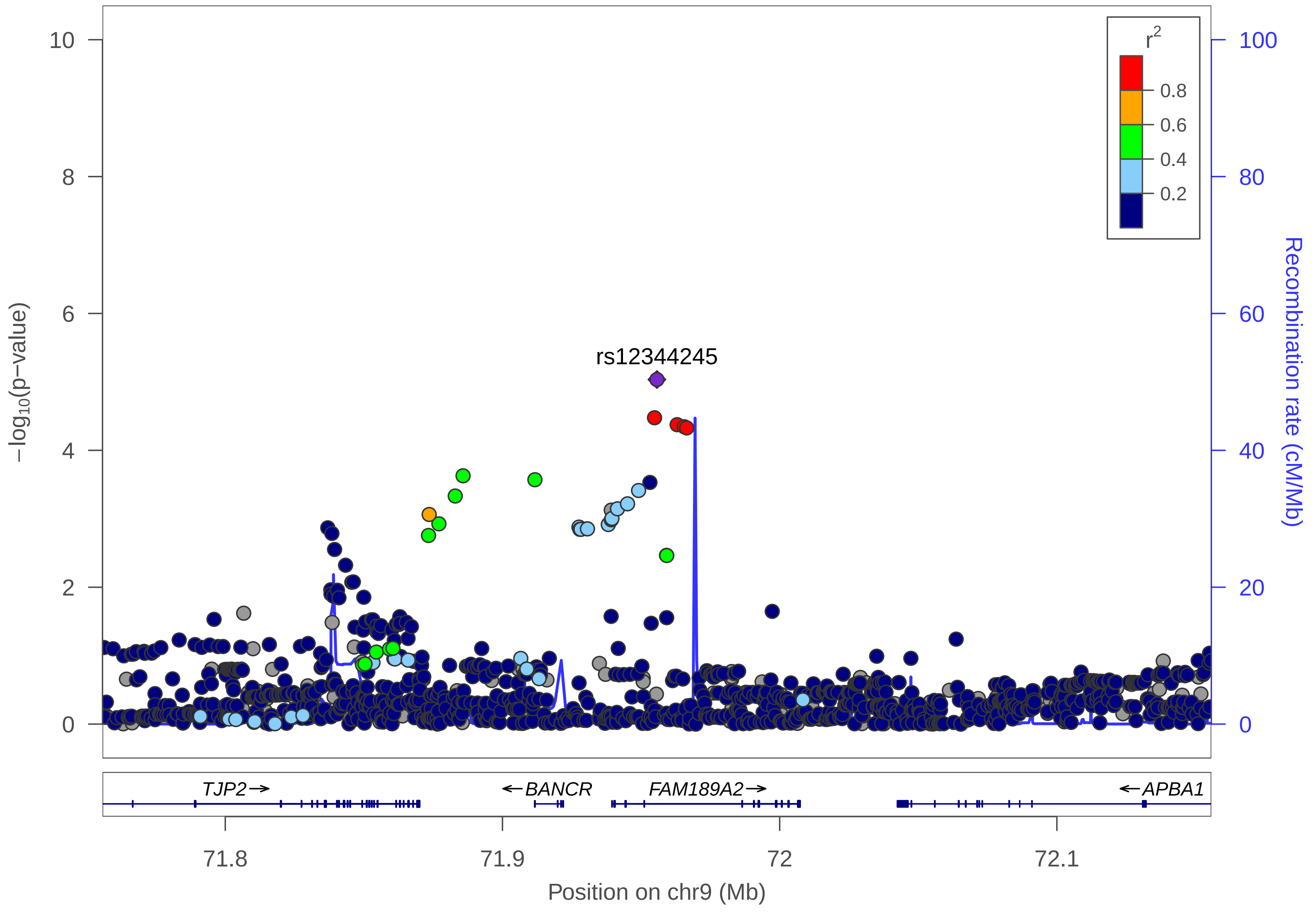

rs12344245
r2
0.8
0.6
0.4
0.2
−log10(p−value)
Recombination rate (cM/Mb)
0
2
4
6
8
10
0
20
40
60
80
100
TJP2→
←BANCR
FAM189A2→
←APBA1
71.8
71.9
72
72.1
Position on chr9 (Mb)

date:	Thu Aug 31 19:43:53 2017
build:	hg19
display range:	chr9:71755717−72155717 [71755717−72155717]
hilite range:	0 − 0 [0 − 0]
reference SNP:	chr9:71955717
number of SNPs plotted:	1218
min P−value:	9.23E−6 [chr9:71955717]
max P−value:	10E−1 [chr9:71969717]

Make more plots at http://csg.sph.umich.edu/locuszoom/
