# Supplementary material for: Novel risk genes identified in a genome-wide association study for coronary artery disease in patients with type 1 diabetes
Source: Cardiovasc Diabetol. 2018 Apr 25;17:61. doi: 10.1186/s12933-018-0705-0 (PMC5916834; doi:10.1186/s12933-018-0705-0)
Supplement: Supplementary file 9 — Additional file 9: Figure S6. Regional association plot at the PKD1 locus. [file 12933_2018_705_MOESM9_ESM.pdf]

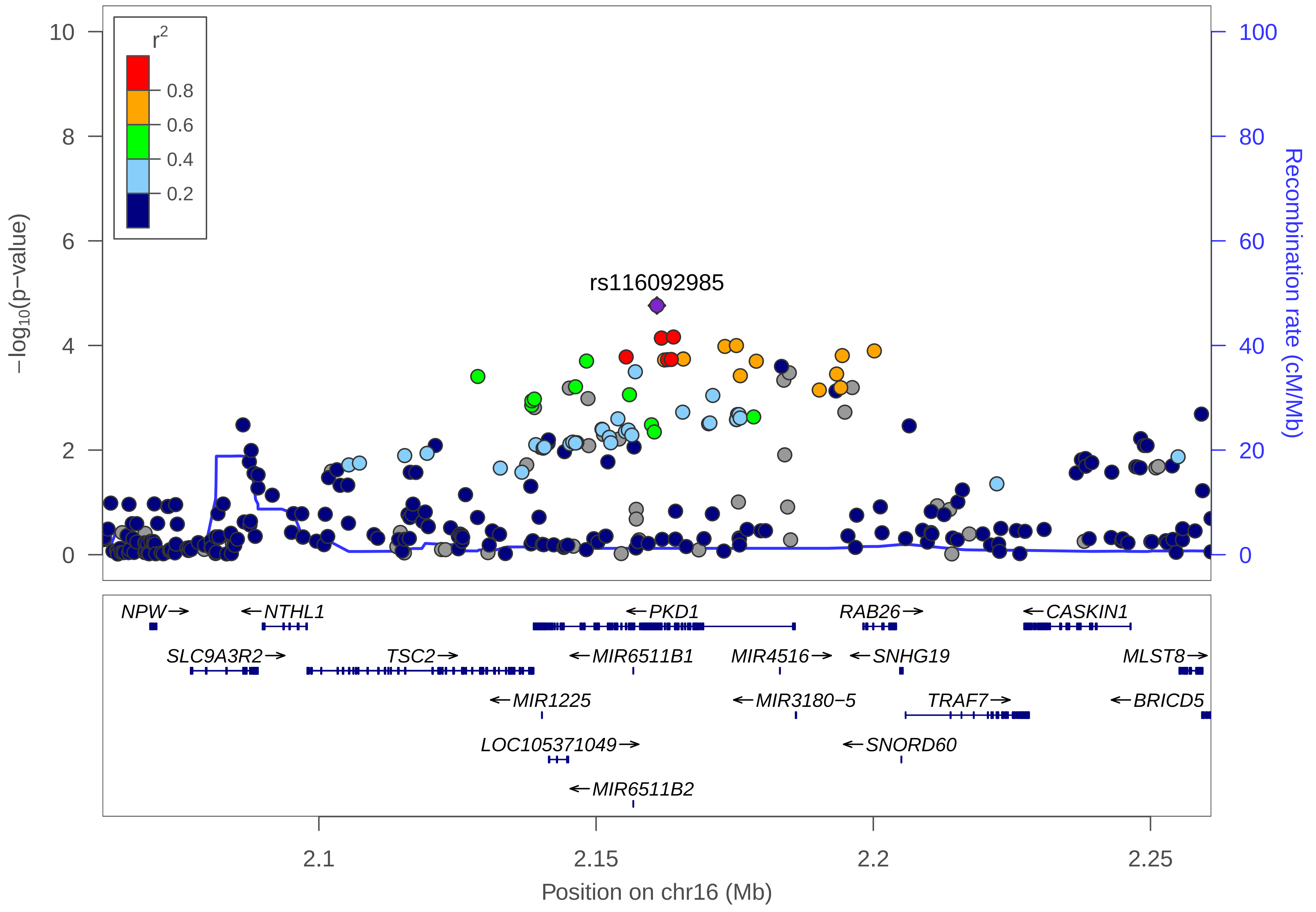

r2
0.8
0.6
0.4
0.2
10
8
6
4
2
0
−log10(p−value)
100
80
60
40
20
0
Recombination rate (cM/Mb)
rs116092985
NPW→
←NTHL1
←PKD1
RAB26→
←CASKIN1
SLC9A3R2→
TSC2→
←MIR6511B1
MIR4516→
←SNHG19
MLST8→
←MIR1225
←MIR3180−5
TRAF7→
←BRICD5
LOC105371049→
←SNORD60
←MIR6511B2
2.1
2.15
2.2
2.25
Position on chr16 (Mb)

date:	Mon Aug 28 19:52:53 2017
build:	hg19
display range:	chr16:2060973−2260973 [2060973−2260973]
hilite range:	0 − 0 [0 − 0]
reference SNP:	chr16:2160973
number of SNPs plotted:	334
min P−value:	1.72E−5 [chr16:2160973]
max P−value:	9.69E−1 [chr16:2063733]

Make more plots at http://csg.sph.umich.edu/locuszoom/
